# *AQP3*-mediated activation of the *AMPK*/*SIRT1* signaling pathway curtails gallstone formation in mice by inhibiting inflammatory injury of gallbladder mucosal epithelial cells

**DOI:** 10.1186/s10020-023-00712-8

**Published:** 2023-08-28

**Authors:** Ganggang Wang, Hao Zhang, Zhijie Zhou, Wenzhi Jin, Xin Zhang, Zenghui Ma, Xiaoliang Wang

**Affiliations:** Department of Hepatobiliary Surgery, Shanghai Pudong Hospital, Fudan University Pudong Medical Center, Shanghai, 201399 China

**Keywords:** *AQP3*, *AMPK*/*SIRT1* signaling pathway, High-throughput sequencing, Cholelithiasis, Gallstone formation, Gallbladder mucosal epithelial cells, Inflammatory injury

## Abstract

**Background:**

Inflammatory injury of gallbladder mucosal epithelial cells affects the development of cholelithiasis, and aquaporin 3 (*AQP3*) is an important regulator of inflammatory response. This study reports a mechanistic insight into *AQP3* regulating gallstone formation in cholelithiasis based on high-throughput sequencing.

**Methods:**

A mouse model of cholelithiasis was induced using a high-fat diet, and the gallbladder tissues were harvested for high-throughput sequencing to obtain differentially expressed genes. Primary mouse gallbladder mucosal epithelial cells were isolated and induced with Lipopolysaccharides (LPS) to mimic an in vitro inflammatory injury environment. Cell biological phenotypes were detected by TdT-mediated dUTP Nick-End Labeling (TUNEL) assay, flow cytometry, Cell Counting Kit-8 (CCK-8) assay, and Trypan blue staining. In addition, enzyme linked immunosorbent assay (ELISA) determined the production of inflammatory factors in mouse gallbladder mucosa.

**Results:**

Whole-transcriptome sequencing data analysis identified 489 up-regulated and 1007 down-regulated mRNAs. Bioinformatics analysis revealed that *AQP3* was significantly down-regulated in mice with cholelithiasis. *AQP3* might also confer an important role in LPS-induced gallbladder mucosal injury. Overexpression of *AQP3* activated the *AMPK* (adenosine monophosphate-activated protein kinase) / *SIRT1* (sirtuin-1) signaling pathway to reduce LPS-induced inflammatory injury of the gallbladder mucosa epithelium, thereby ameliorating gallbladder damage and repressing gallstone formation in mice.

**Conclusion:**

Data from our study highlight the inhibitory role of *AQP3* in gallbladder damage and gallstone formation in mice by reducing inflammatory injury of gallbladder mucosal epithelial cells, which is achieved through activation of the *AMPK*/*SIRT1* signaling pathway.

**Supplementary Information:**

The online version contains supplementary material available at 10.1186/s10020-023-00712-8.

## Introduction

Asymptomatic or symptomatic gallstones grow inside the gallbladder or bile duct, and those with symptoms or complications indicate gallstone disease (Lammert et al. 2016). Cholelithiasis is mainly classified into two types, namely cholesterol and pigment gallstones (Chen et al. 2022). Inflammation is a pivotal mediator in forming gallstones (Maurer et al. 2007; O’Leary et al. 2022). In this context, seeking novel targets for treating cholelithiasis based on inflammation control is significant.

It should be noted that aquaporin 3 (*AQP3*) was screened as the important differentially expressed gene (DEG) in cholelithiasis. *AQP3* is an integral membrane protein that promotes water and glycerol transport across cell membranes (Lim et al. 2016). AQPs can facilitate the transepithelial water transport in the gallbladder and regulate bile composition (Li et al. 2009). AQPs have been indicated to be involved in multiple diseases, including gallstone disease (Jeyaseelan et al. 2006). However, the role and specific mechanism of *AQP3* in cholelithiasis remains unclear.

Interestingly, we further found that *AQP3* may modulate the adenosine monophosphate-activated protein kinase (*AMPK*)/sirtuin-1 (*SIRT1*) signaling pathway in lipopolysaccharide (LPS)-induced gallbladder mucosal epithelial cells. *AMPK* is responsible for multiple metabolic pathways for sustaining appropriate levels of intracellular adenosine triphosphate upon energetic and/or cellular stress (Wu & Zou, 2020), and *SIRT1*, a class-III histone deacetylase, is implicated in gene regulation, maintenance of genome stability, and cell functions (Alves-Fernandes & Jasiulionis, 2019). Of note, activating the *AMPK*/*SIRT1* signaling pathway could alleviate inflammatory injury of epithelial cells (Li et al. 2019; Zhang & Wu, 2021). Furthermore, leptin-mediated *AMPK*α2 regulated bile acid metabolism, thereby affecting cholelithiasis, especially the formation of primary intrahepatic bile duct stones (Wen et al. 2021). In addition, hepatic deprivation of *SIRT1* was unfolded to induce cholesterol gallstone formation in a mouse model (Purushotham et al. 2012). Therefore, modulation of the *AMPK*/*SIRT1* signaling pathway to ameliorate inflammatory injury may be one of the potential therapeutic tools to repress cholelithiasis.

Our study aims to investigate the possible molecular mechanism of *AQP3* in regulating cholelithiasis, involved with regulating the *AMPK*/*SIRT1* signaling pathway, in the hope of finding novel directions for repressing and treating cholelithiasis.

## Materials and methods

### Ethical approval

The study was conducted under the approval of the Ethics Committee of Shanghai Pudong Hospital (No. QWJWLX-01). All animal experiment procedures were conducted per the *Guide for the Care and Use of Laboratory Animals*.

### Induction of mouse model of cholelithiasis

Five neonatal C57BL/6J mice, each weighing between 2 and 5 g and aged from 2 to 3 days, along with forty adult male C57BL/6J mice, each weighing between 20 and 30 g and aged between 6 and 8 weeks, were procured from Vital River Laboratory Animal Technology Co., Ltd., located in Beijing, China. These mice were subsequently housed in individual cages within a laboratory environment free of pathogens. The health of the mice was monitored throughout a week of acclimatization before the commencement of the study (Lu et al. 2022).

A mouse model of cholelithiasis was developed using a high-fat diet induction method. Following a week of acclimatization, the model construction began with 6–8 week old male C57BL/6J mice. Ten mice were in the control group and fed with standard chow (LAD0020, Trophic Animal Feed High-tech Co., Ltd., Nantong, Jiangsu, China). The remaining 30 mice were used for the model development: all mice in the model group were fed daily with a high-fat diet (TP 06136F4, Trophic Animal Feed High-tech Co., Ltd., Nantong, Jiangsu, China) that included 15% butterfat, 1% cholesterol, 0.5% cholic acid, 2% corn oil, 50% sucrose, 20% casein, and essential minerals and vitamins. This feeding routine was maintained for 8 weeks, after which the mice were euthanized. The gallbladder was retrieved from below the liver during the necropsy. The gallbladder was opened to visually inspect for macroscopic crystalline stones. Tissue samples, including the gallbladder, were collected for subsequent experimental analysis. Additionally, a portion of the tissue was rapidly frozen in liquid nitrogen and stored at -80 °C, making it readily available for other analyses (Moschetta et al. 2004; He et al. 2021; Hu et al. 2022).

### High-throughput sequencing

Gallbladder tissue samples were collected from three normal mice and three mice with cholelithiasis. Total RNA was isolated from these six samples using the total RNA isolation kit (12,183,555, Invitrogen, USA). The amount of total RNA was quantified using Nanodrop, and the integrity of this RNA was evaluated by agarose gel electrophoresis. High-quality total RNA was reverse-transcribed into cDNA. A cDNA library was constructed using the TruSeq RNA Sample Preparation Kit (Illumina, San Diego, CA, USA), and sequencing was performed on the Illumina NextSeq 500 platform. The raw image data obtained from sequencing were converted to raw reads via base calling. To control the quality of raw reads, sequencing adapter sequences were removed from raw reads using cutadapt, and low-quality sequences were filtered out. The remaining reads are referred to as “clean reads.“ The clean reads were aligned to the mouse reference genome using Hisat2 software. Gene expression was quantified using the R software package, generating a gene expression matrix (Hu et al. 2022).

### Acquisition and functional analysis of DEGs

Differential gene expression was analyzed using the “edgeR” package in R with a screening threshold of |log2FC| > 1 (FC: foldchange) and p < 0.05 to identify differentially expressed genes. The “clusterProfiler” package in R was used for GO (Gene Ontology) and KEGG (Kyoto Encyclopedia of Genes and Genomes) analyses. The top 100 differentially expressed genes with the highest |log2FC| values were input into the STRING website (https://cn.string-db.org/), with a confidence value 0.96, to obtain a PPI (Protein-Protein Interaction) network. The “tsv” file from the STRING website was obtained, and proteins were ranked according to the number of connections (degree value) between proteins. Genes with the highest degree values, hub genes, were selected. The R package and Cytoscape software (v3.9.1) generated bar charts of the hub genes and downstream signaling factors (Movahed et al. 2021).

### Isolation and characterization of primary mouse gallbladder mucosal epithelial cells

Primary cultures of gallbladder mucosal epithelial cells were isolated from 5 C57BL/6J mice aged 2–3 days (2–5 g). In brief, the mice were sacrificed and dissected. The gallbladder was excised from the liver and sectioned into 3–4 fragments. These gallbladder fragments were directly embedded on a collagen gel plate with 3 ml of culture medium. Collagen protein gel plates were made with 60 mm culture dishes and 3 ml collagen protein solution, then incubated at 37 ˚C. The collagen protein solution was prepared using 0.3% collagen acid solution (C3867, Merck, Germany), 10x Hank’s Balanced Salt Solution (HBSS, H1641, Sigma-Aldrich, St. Louis, MO, USA), and 0.8 N NaOH (8:1:1) (S5881, Sigma-Aldrich, St. Louis, MO, USA). The culture medium consisted of Dulbecco’s modified Eagle medium/HamF12 (DMEM/Ham F12, D0547, Gibco, USA) supplemented with 10% FBS (12,483,020, Gibco, USA) and 1% double antibiotics. The culture medium was generally changed every 2–3 days. Cellular proliferation from fragments was evident during the cultivation process, demonstrating a cobblestone-like appearance. After 5–7 days of cultivation, fragments in the medium were removed under a microscope using sterile tweezers. Most of the gallbladder mucosal epithelial cells that spread on the gel could be obtained through this process, as the epithelial cells expand horizontally on the gel while fibroblasts grow within the gel. Therefore, most of the epithelial cells on the surface of the gel were collected.

Subsequently, cells were collected by centrifugation at 500 g/min for 5 min at 4 ℃ and resuspended in Dulbecco’s modified Eagle medium/HamF12 culture medium supplemented with 10% fetal bovine serum and 1% double antibiotics. When the cell density reached 70%, the cell type and purity were identified by detecting the fluorescence signal of CD326 (Epithelial Cell Adhesion Molecule: EpCAM) antibody (1:20, 11-5791-82, Thermo Fisher Scientific, USA) using immunofluorescence. The cell purity was confirmed to be 90%. Then, cells were continuously passaged and cultured for 3–4 generations before performing subsequent experimental procedures (Imamura et al. 2017).

### Lentiviral transduction of primary gallbladder mucosal epithelial cells

The *AQP3* expression plasmid and its associated lentivirus were obtained from Hanheng Biotechnology Co., Ltd. (Shanghai, China). When the cell confluence reached 50%, the cells were infected with the lentivirus (10 MOI), and then 5 µg/mL puromycin was added to the culture medium. After culturing for 3 days, a stably transfected cell line was established (Liu et al. 2019).

### LPS-induced inflammatory injury model of gallbladder mucosal epithelial cells

In vitro gallbladder mucosal epithelial inflammatory cell injury model was established as follows: 200 µg/mL LPS (L2630, Sigma-Aldrich, St. Louis, MO, USA) was added to the gallbladder mucosal epithelial cells in a logarithmic growth phase in each group and stimulated for 24 h (Lali et al. 2000; Zhao et al. 2015). The in vitro cell experiments were divided into the following groups: Control group (primary gallbladder mucosal epithelial cells), LPS group (primary gallbladder mucosal epithelial cells treated with LPS for 24 h), LPS + oe-NC group (primary gallbladder mucosal epithelial cells stably transfected with overexpression negative control plasmid and treated with LPS for 24 h), LPS + oe-*AQP3* group (primary gallbladder mucosal epithelial cells stably transfected with *AQP3* overexpression plasmid and treated with LPS for 24 h), and LPS + oe-*AQP3* + *AMPK* inhibitor group (primary gallbladder mucosal epithelial cells stably transfected with *AQP3* overexpression plasmid, and treated with LPS and *AMPK* inhibitor for 24 h). The *AMPK* inhibitor was Dorsomorphin (at a concentration of 10 µM, S7840, Selleck) (Mattos et al. 2022). oe: overexpression; NC: negative control.

### TUNEL(TdT-mediated dUTP Nick-End labeling) staining and flow cytometry for cell apoptosis

To assess cell apoptosis induced by LPS, a TUNEL Cell Apoptosis Assay Kit (C1089, Beyotime, Shanghai, China) was used. Gallbladder mucosal epithelial cells were fixed with paraformaldehyde and then incubated with TUNEL solution for 30 min. After staining with DAPI for 5 min, the cells were imaged using a fluorescence microscope. Image J software was used to analyze cell apoptosis by calculating the number of TUNEL-positive cells.

Apoptosis was further examined using the Annexin V-FITC/PI Cell Apoptosis Detection Kit (C1062L, Beyotime, Shanghai, China). In brief, cells were washed and resuspended with PBS after LPS treatment. Approximately 1 × 10^5^ gallbladder mucosal epithelial cells were stained in the dark with 10 µL of Annexin V-Fluorescein Isothiocyanate (FITC) and 5 µL of Propidium Iodide (PI), followed by apoptosis detection with a flow cytometer (BD Biosciences, Franklin Lakes, NJ, USA) (Lu et al. 2022).

### CCK-8 (cell counting Kit-8) assay

Cell viability was evaluated using a CCK-8 assay kit (C0037, Beyotime, Shanghai, China). Following LPS induction, cells were washed and resuspended with PBS, then added to a 96-well plate (2 × 10^3^ cells/well). Next, 10 µL of CCK-8 solution was added to each well and incubated at 37 ˚C in 5% CO_2_ for 4 h. Absorbance at 450 nm was measured using a microplate reader (E0227, Beyotime, Shanghai, China) (Liu et al. 2011).

### Trypan blue staining

Gallbladder epithelial cells were mixed with Trypan Blue Stain Solution (C0011, Beyotime, Shanghai, China). After 3 min, dishes containing live (colorless cytoplasm) and dead cells (blue cytoplasm) were counted. The positivity rate of Trypan Blue was quantified using Image J software across eight randomly selected fields of view (Huang et al. 2010).

### In vivo animal experiment

The high-fat diet-fed model mice were divided into three groups: one served as the model group, with no treatment; the other two model groups were treated with oe-NC and oe-*AQP3* lentivirus infections, respectively. Specifically, 4 ug of lentivirus was dissolved in 400 µL of 0.9% NaCl and injected once every two weeks via the mouse tail vein for four injections. The in vivo animal experiment groups were: Normal group, Model group, oe-NC group, and oe-*AQP3* group, with 10 mice in each group. After 8 weeks, mice were euthanized, and the gallbladder was removed from beneath the liver. The gallbladder was cut open to check for visible crystalline stones. Simultaneously, mouse gallbladder and other tissues were collected for subsequent related experimental testing. Furthermore, some tissues were rapidly frozen in liquid nitrogen and stored at -80 °C for future analysis (Dalsgaard et al. 2018; Hu et al. 2022).

### Preparation of gallbladder tissue sections

Upon euthanization of mice from each group, the entire gallbladder was excised and fixed in 4% paraformaldehyde for 24 h, then sequentially dehydrated in 20% and 30% sucrose solutions. Tissue samples were embedded in the Optimal Cutting Temperature compound (OCT), and 10 µM longitudinal sections were cut using a cryostat (Leica Microsystems Inc., Buffalo Grove, IL, USA) (Lu et al. 2022).

### H&E (hematoxylin-eosin staining) staining

Gallbladder tissues were collected from each group of mice, fixed in 10% neutral buffered formalin, then embedded in paraffin for sectioning. The sections were deparaffinized in xylene. The sections were then stained with hematoxylin, washed with distilled water, immersed in 95% ethanol, stained with eosin, hydrated through gradient alcohol, dehydrated with xylene, air-dried, and finally mounted with neutral resin for observation under a light microscope. The primary observations were made on the pathological conditions of gallbladder mucosal tissue, including the thickness of the gallbladder wall, the extent of matrix cell infiltration, and the degree of mucosal damage. These evaluations assessed the pathological conditions of the gallbladder mucosal tissues in mice (Moschetta et al. 2004; Wei et al. 2019).

### TUNEL staining

Apoptosis was measured using a TUNEL Cell Apoptosis Detection Kit (C1089, Beyotime Biotechnology, Shanghai, China). Gallbladder frozen sections previously prepared were fixed in 4% paraformaldehyde at 20 ˚C for 30 min and then washed thrice with PBS (phosphate buffered saline). Sections were incubated at room temperature for 5 min with PBS solution of ImmunoStaining Enhancer (P0097, Beyotime Biotechnology, Shanghai, China), followed by three washes with PBS. Afterward, they were incubated overnight at 4 ˚C with anti-CD326 antibody (1:20, 11-5791-82, Thermo Fisher Scientific, USA). After three washes with PBS, sections were incubated at room temperature for 2 h with Alexa Fluor 488-labeled secondary antibody (ab150077, 1:200, Abcam, UK). TUNEL detection solution was prepared per the instruction manual, adding 5 µL TdT enzyme, 45 µL fluorescent labeling solution, and 50 µL TUNEL detection solution to each sample, followed by thorough mixing. This mixture was added to the samples and incubated at 37 ℃ in the dark for 60 min. After three washes with PBS, sections were mounted with Anti-fade Mounting Medium with DAPI (4’,6-diamidino-2-phenylindole) (P0131, Beyotime Biotechnology, Shanghai, China). Under a fluorescence microscope, the gallbladder mucosal epithelial cells undergoing apoptosis were co-located by green and red fluorescence, while the normal gallbladder mucosal epithelial cells were co-located by blue and red fluorescence (Huang et al. 2020).

### ELISA (enzyme linked immunosorbent assay)

Mouse gallbladder tissues or cultured gallbladder mucosal epithelial cells were collected and dispersed. After lysis and centrifugation, the supernatant was collected. According to the methods provided by the kit supplier (Beyotime Biotechnology, Shanghai, China), the levels of TNF-α (PT512), IL-1β (PI301), and IL-18 (PI553) were determined by enzyme-linked immunosorbent assay (ELISA) (Guo et al. 2022).

### RT-qPCR and Western blot

Firstly, total RNA was extracted from gallbladder mucosal epithelial cells and gallbladder tissues (Maurya et al. 2018). The tissues were homogenized using TRIzol reagent (10,296,010, Thermofisher, USA) at a ratio of 1 mL per 100 mg of tissue. After adding chloroform (200 µL) and mixing thoroughly, the mixture was centrifuged at 12,000 g for 10 min at 4 °C. The aqueous phase was collected and mixed with isopropanol (500 µL) to precipitate RNA. The RNA was then dissolved in RNase-free water (10–30 µL) and quantified using a Nanodrop spectrophotometer (Nanodrop 3300, Thermofisher, USA). Reverse transcription was performed on 1 µg of total RNA using the TaqMan Reverse Transcription reagent (N8080234, Thermofisher, USA). PCR analysis was conducted using the PowerUp SYBR Green premix reagent kit (A25741, Thermofisher, USA). GAPDH was used as an internal reference, and the relative expression levels of each gene were analyzed using the 2-ΔΔCT method (Peng et al. 2020; Franke et al. 2021). The sequences of the primers are shown in Table S1.

Total protein from cells and cortical tissues was extracted using RIPA (Radio Immunoprecipitation Assay) lysis buffer containing 4% protease inhibitor (P0013B, Beyotime, Shanghai, China) according to the instructions (1 mL per 100 mg of tissue). The protein concentration was determined using the BCA (bicinchoninic acid) Protein Assay Kit (P0010S, Beyotime). Equal amounts of protein samples were separated on a sodium dodecyl sulfate-polyacrylamide gel (SDS-PAGE) and then transferred onto PVDF (polyvinylidene difluoride) membranes (FFP24, Beyotime) using the wet transfer method. The membranes were blocked with TBST (Tris Buffered Saline with Tween ® 20) containing 5% skim milk (P0216-300 g, Beyotime) at room temperature for 1 h and then incubated with primary antibodies overnight at 4 °C. After washing with TBST, the membranes were incubated with horseradish peroxidase-conjugated goat anti-rabbit (A0208, Beyotime) or goat anti-mouse secondary antibodies (A0216, Beyotime) at room temperature for 1 h. The manufacturer information of primary antibodies is provided in Table S2. The membranes were then developed using the ECL (Enhanced Chemiluminescent) Chemiluminescent Substrate Reagent Kit (P0018FS, Beyotime), and the bands were visualized using the ECL chemiluminescent detection system (ChemiDoc XRS+, Bio-Rad). Protein quantification was conducted using Image J software, with the grayscale value of each protein relative to the internal control GAPDH used for protein quantification (Gong et al. 2017). Each experiment was repeated three times.

### Statistical analysis

All data were analyzed using SPSS 21.0 statistical software (IBM, USA). Quantitative data were presented as the mean ± standard deviation. The t-test was used to compare two groups, and one-way analysis of variance (one-way ANOVA) was used for multiple group comparisons. A p-value of less than 0.05 was considered statistically significant.

## Results

### *AQP3* was significantly downregulated in mice with cholelithiasis

**Fig. 1:**
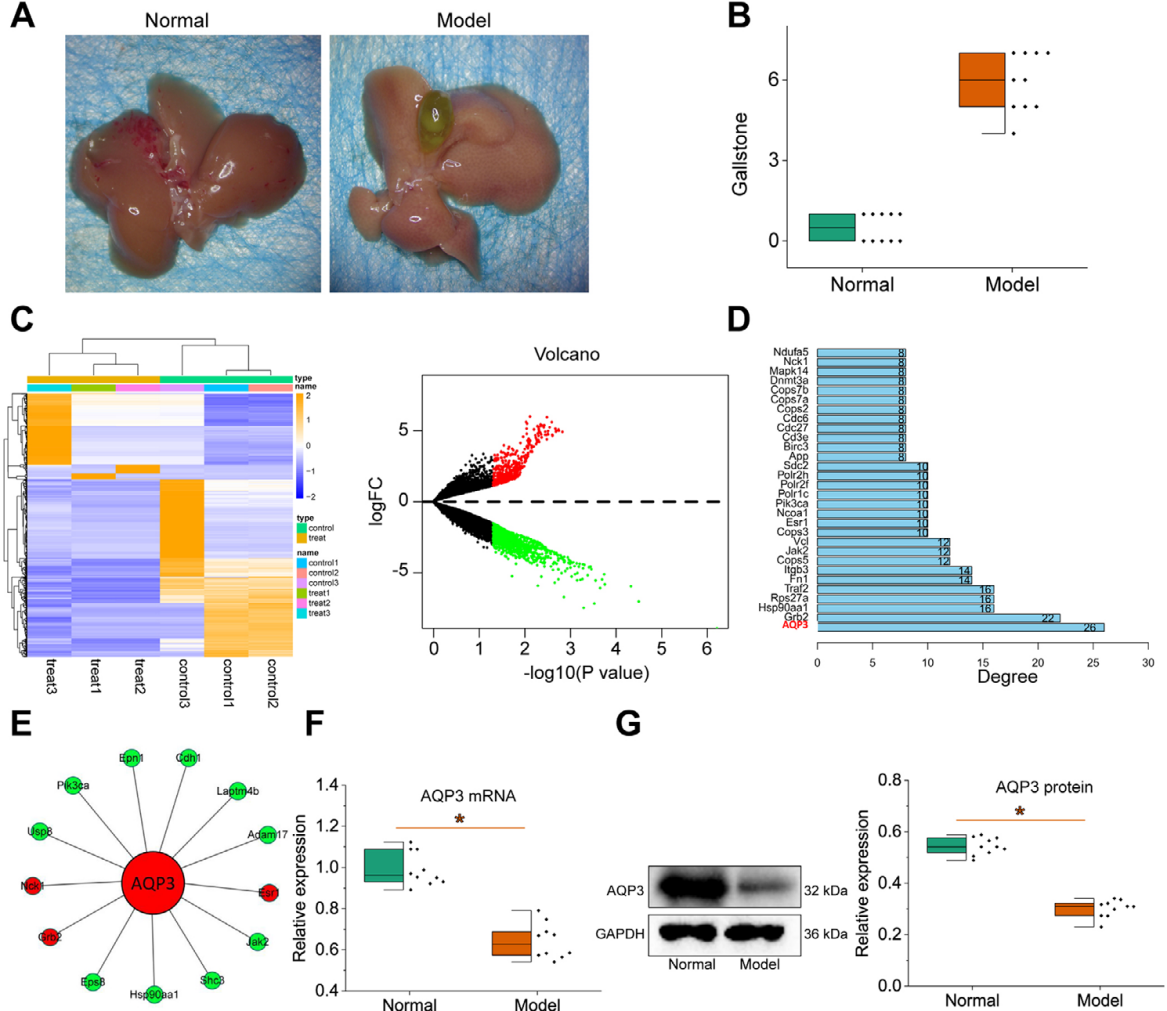
High-throughput sequencing and *AQP3* expression analysis of gallbladder tissues from cholelithiasis and normal control mice. **A**, Image of gallbladder stones in cholelithiasis mice; **B**, Gallbladder stone score; **C**, Heatmap and volcano plot of differentially expressed genes in gallbladder tissues of normal control and cholelithiasis mice detected by high-throughput sequencing. Green indicates downregulation, red indicates upregulation; **D**, Bar chart of hub genes in the differentially expressed gene PPI network between normal control and cholelithiasis mice identified by high-throughput sequencing; **E**, PPI protein interaction network of the hub gene *AQP3* in gallbladder tissues of normal control and cholelithiasis mice identified by high-throughput sequencing. Green indicates downregulation, red indicates upregulation; **F**, RT-qPCR analysis of *AQP3* mRNA expression in gallbladder tissues of normal control and cholelithiasis mice; **G**, Western blot analysis of *AQP3* protein expression in gallbladder tissues of normal control and cholelithiasis mice. *p < 0.05, vs. normal control

Compared to the normal control group, the incidence of gallstones in mice with cholelithiasis is significantly higher (Fig. 1A-B). Therefore, high-throughput sequencing was performed on three gallbladder samples from the normal control group and the cholelithiasis model group, identifying 1496 differentially expressed genes. Among them, 489 genes were significantly up-regulated, and 1007 genes were significantly downregulated in the cholelithiasis model group (Fig. 1C).

The top 100 differentially expressed genes with the highest |log2FC| values were selected using the STRING database to construct a protein-protein interaction (PPI) network. Then, the proteins were ranked based on the number of connections (degree value) with other proteins, and the genes with the highest degree value, also known as hub genes, were selected. Finally, the regulatory network was visualized using the Cytoscape software, identifying 30 hub genes (Fig. 1D) and 14 differentially expressed genes up or down-regulated concerning the key hub gene *AQP3* (Fig. 1E). The top 10 hub genes ranked by degree value were *AQP3*, GRB2, HSP90AA1, RPS27A, TRAF2, FN1, ITGB3, COPS5, TGF-β, and VCL. Previous studies have suggested that the Aquaporins (AQPs) family plays a role in the injury and regeneration of gallbladder epithelial cells (Mobasheri & Marples, 2004; Li et al. 2009). Therefore, we suggest that *AQP3* may be important in LPS-induced gallbladder mucosal damage.

To verify whether the changes of *AQP3* in the gallbladder tissues of mice with cholelithiasis were consistent with the sequencing results, we examined the expression of *AQP3* in the gallbladder tissues of normal control mice and mice with cholelithiasis. RT-qPCR and Western blot experiments showed that *AQP3* was significantly down-regulated in the gallbladder tissues of mice with cholelithiasis (Fig. 1F-G).

### Low *AQP3* expression might be associated with inflammatory injury of gallbladder mucosal epithelial cells in mice with cholelithiasis

**Fig. 2:**
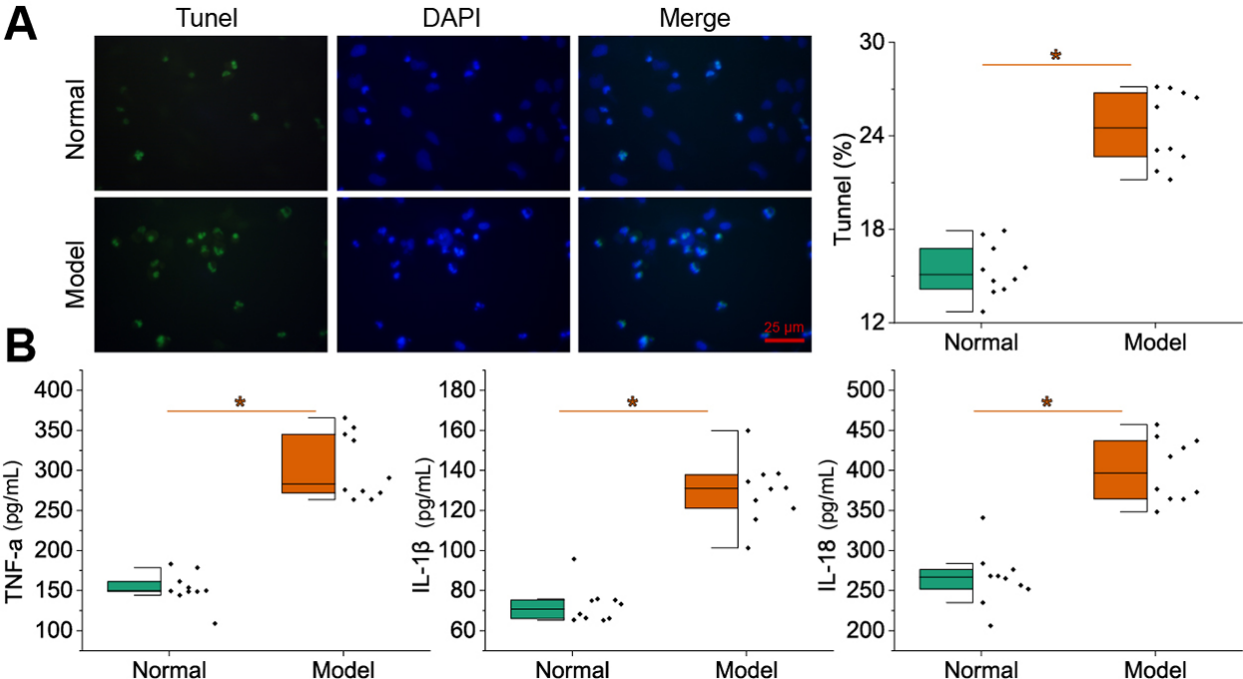
Damage status of gallbladder mucosal epithelial cells and detection of cell injury markers in mice with cholelithiasis. **A**, TUNEL staining to detect inflammatory injury of gallbladder mucosal epithelial cells of normal control mice and mice with cholelithiasis. **B**, ELISA to detect the expression of TNF-α, IL-1β, and IL-18 in gallbladder tissues of normal control mice and mice with cholelithiasis. n = 10. * *p* < 0.05

To investigate whether *AQP3* was associated with LPS-induced inflammatory injury of gallbladder mucosal epithelial cells, we examined the injury of gallbladder mucosal epithelial cells in mice with cholelithiasis. As shown in Fig. 2A, *AQP3* was poorly expressed in the mucosal tissues of mice with cholelithiasis. We further found by TUNEL staining that there were notably more TUNEL-positive cells in the gallbladder mucosa tissue of mice with cholelithiasis than in the normal control mice (Fig. 2B). In addition, the ELISA results showed that TNF-α, IL-1β, and IL-18 expression was markedly up-regulated in the gallbladder tissues of mice with cholelithiasis compared with the normal control mice.

The above results indicated that there was inflammatory injury of the gallbladder mucosa epithelium in mice with cholelithiasis and that the low expression of *AQP3* might be related to the inflammatory injury of the gallbladder mucosal epithelial cells in mice with cholelithiasis.

### Overexpression of *AQP3* inhibited LPS-induced inflammatory injury of gallbladder mucosal epithelial cells

**Fig. 3:**
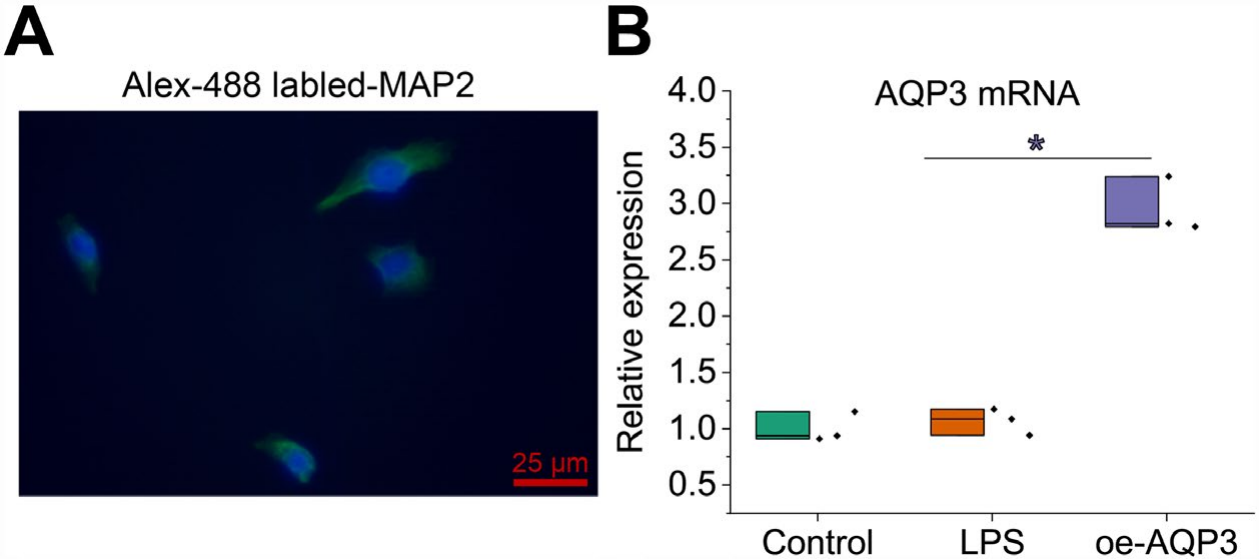
Purity of primary gallbladder mucosal epithelial cells and lentiviral transduction. **A**, Immunofluorescence detection of the purity of primary mouse gallbladder mucosal epithelial cells. **B**, RT-qPCR to detect *AQP3* overexpression efficiency in primary gallbladder mucosal epithelial cells. Cell experiments were repeated three times. * *p* < 0.05

Next, to observe the effect of *AQP3* on LPS-induced inflammatory injury of gallbladder mucosal epithelial cells, we obtained primary gallbladder mucosal epithelial cells stably overexpressing *AQP3* by primary cell culture (cell purity > 95%) and cell transfection (Fig. 3A). Furthermore, *AQP3* mRNA expression in gallbladder mucosal epithelial cells was significantly lower after LPS induction, while this expression rebounded after further treatment with oe-*AQP3* (Fig. 3B).

**Fig. 4:**
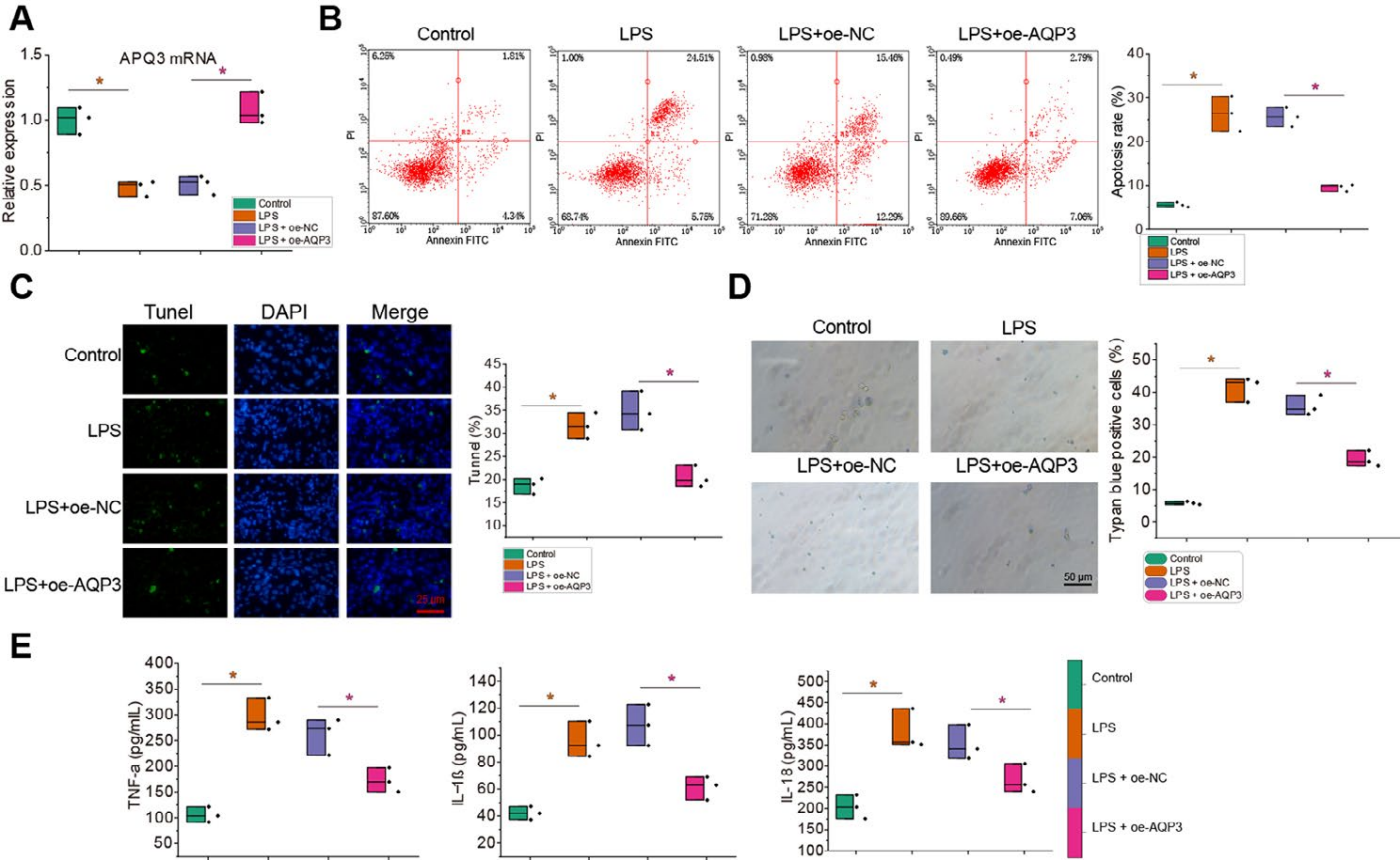
Effects of *AQP3* on LPS-induced inflammatory injury of gallbladder mucosal epithelial cells. **A** RT-qPCR to detect the mRNA expression of *AQP3* in gallbladder mucosal epithelial cells. **B**, Flow cytometry to detect the inflammatory injury of gallbladder mucosal epithelial cells. **C**, immunofluorescence to detect the inflammatory injury of gallbladder mucosal epithelial cells. Red fluorescence represents TUNEL, and blue fluorescence represents the nucleus. **D**, Trypan blue staining to observe the pyroptosis of gallbladder mucosal epithelial cells. **E**, ELISA to detect the expression of TNF-α, IL-1β, and IL-18 in gallbladder mucosal epithelial cells. All cell experiments were repeated 3 times. * *p* < 0.05

Subsequently, RT-qPCR detection confirmed the successful overexpression of *AQP3* (Fig. 4A). Cell injury was examined, and the results showed that LPS induction resulted in massive apoptosis of gallbladder mucosal epithelial cells and increases in cell death rate, accompanied by marked increases in TNF-α, IL-1β, and IL-18 expression; further overexpression of *AQP3* could reverse all the above changes of in LPS-induced gallbladder mucosal epithelial cells (Fig. 4B-E).

These results suggested the inhibitory role of overexpression of *AQP3* in LPS-induced inflammatory injury of gallbladder mucosal epithelial cells.

### *AQP3* activated the *AMPK*/*SIRT1* signaling pathway in LPS-induced gallbladder mucosal epithelial cells

We searched existing literature to further investigate the molecular mechanism by which *AQP3* could attenuate LPS-induced inflammatory injury of gallbladder mucosal epithelial cells. We found that inhibition of *AQP3* expression can further inhibit the activation of the *AMPK* pathway (Li et al. 2009). In addition, many studies have shown that activation of the *AMPK*/*SIRT1* signaling pathway can reduce inflammatory injury of gallbladder mucosal epithelial cells (Li et al. 2019; Zhang & Wu, 2021). Therefore, we hypothesized that *AQP3* might reduce inflammatory injury of gallbladder mucosal epithelial cells by activating *AMPK*/*SIRT1* signaling pathway.

**Fig. 5:**
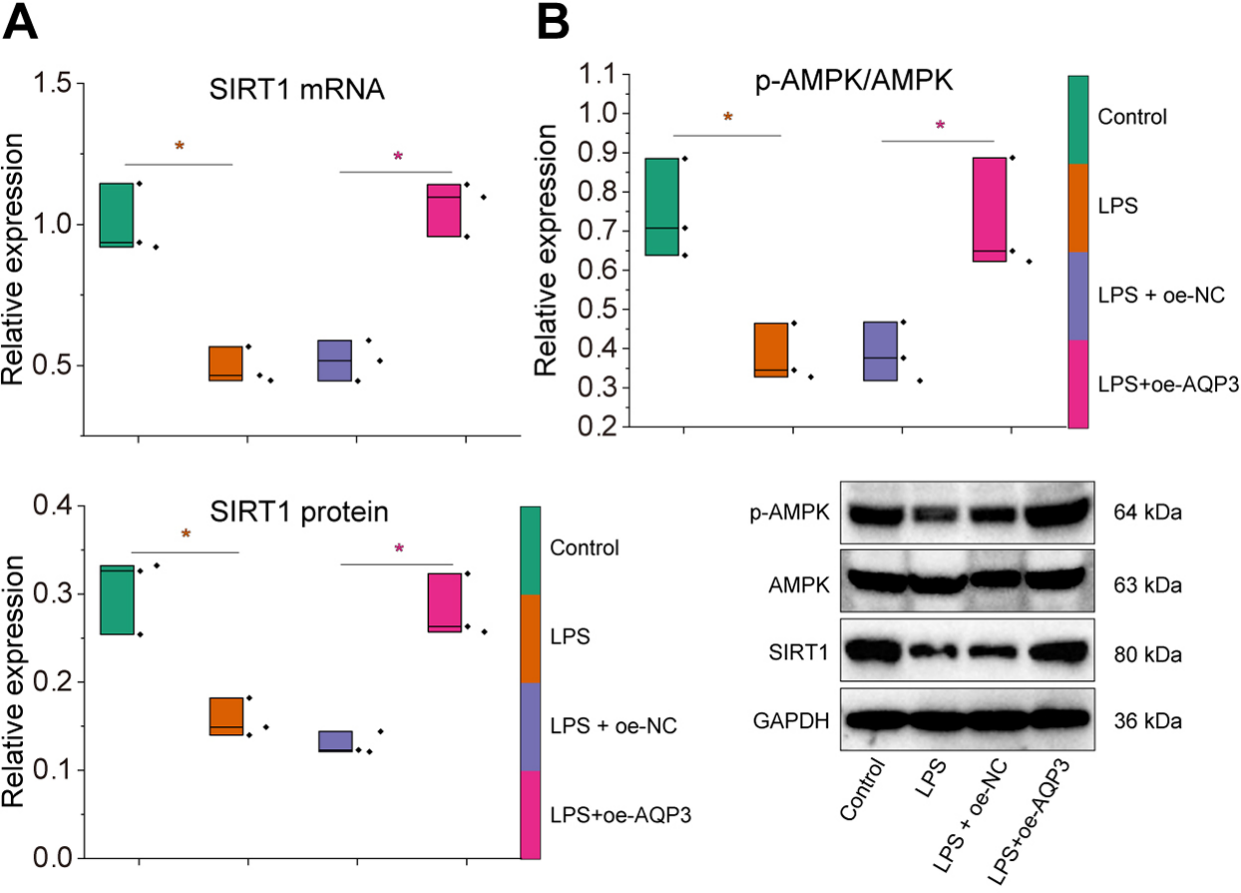
Effects of *AQP3* on *AMPK*/*SIRT1* signaling pathway in LPS-induced gallbladder mucosal epithelial cells. **A** RT-qPCR to detect the mRNA expression of *SIRT1* in LPS-induced gallbladder mucosal epithelial cells after *AQP3* overexpression. **B**, Western blot to detect the protein expression and phosphorylation level of *AMPK* and protein expression of *SIRT1* in LPS-induced gallbladder mucosal epithelial cells after *AQP3* overexpression. All cell experiments were repeated three times. * *p* < 0.05

Therefore, we examined the expression of *AMPK* and *SIRT1* in gallbladder mucosal epithelial cells. The *SIRT1* mRNA levels were significantly downregulated, and phosphorylation levels of *AMPK* were decreased in gallbladder mucosal epithelial cells after LPS induction. Further overexpression of *AQP3* could promote the mRNA levels of *AMPK* and *SIRT1*, protein expression and phosphorylation level of *AMPK* and protein expression of *SIRT1* in LPS-induced gallbladder mucosal epithelial cells (Fig. 5A, B).

*AQP3* could activate the *AMPK*/*SIRT1* signaling pathway in LPS-induced gallbladder mucosal epithelial cells.

### Overexpression of *AQP3* attenuated LPS-induced inflammatory injury of gallbladder mucosal epithelial cells through activation of the *AMPK*/*SIRT1* signaling pathway

The study moved to further investigate whether the inhibitory effect of *AQP3* on LPS-induced inflammatory injury of gallbladder mucosal epithelial cells was related to the *AMPK*/*SIRT1* signaling pathway. We used the *AMPK* inhibitor Dorsomorphin based on overexpression of *AQP3* to explore the link between *AQP3* and the *AMPK*/*SIRT1* signaling pathway under LPS conditions.

**Fig. 6:**
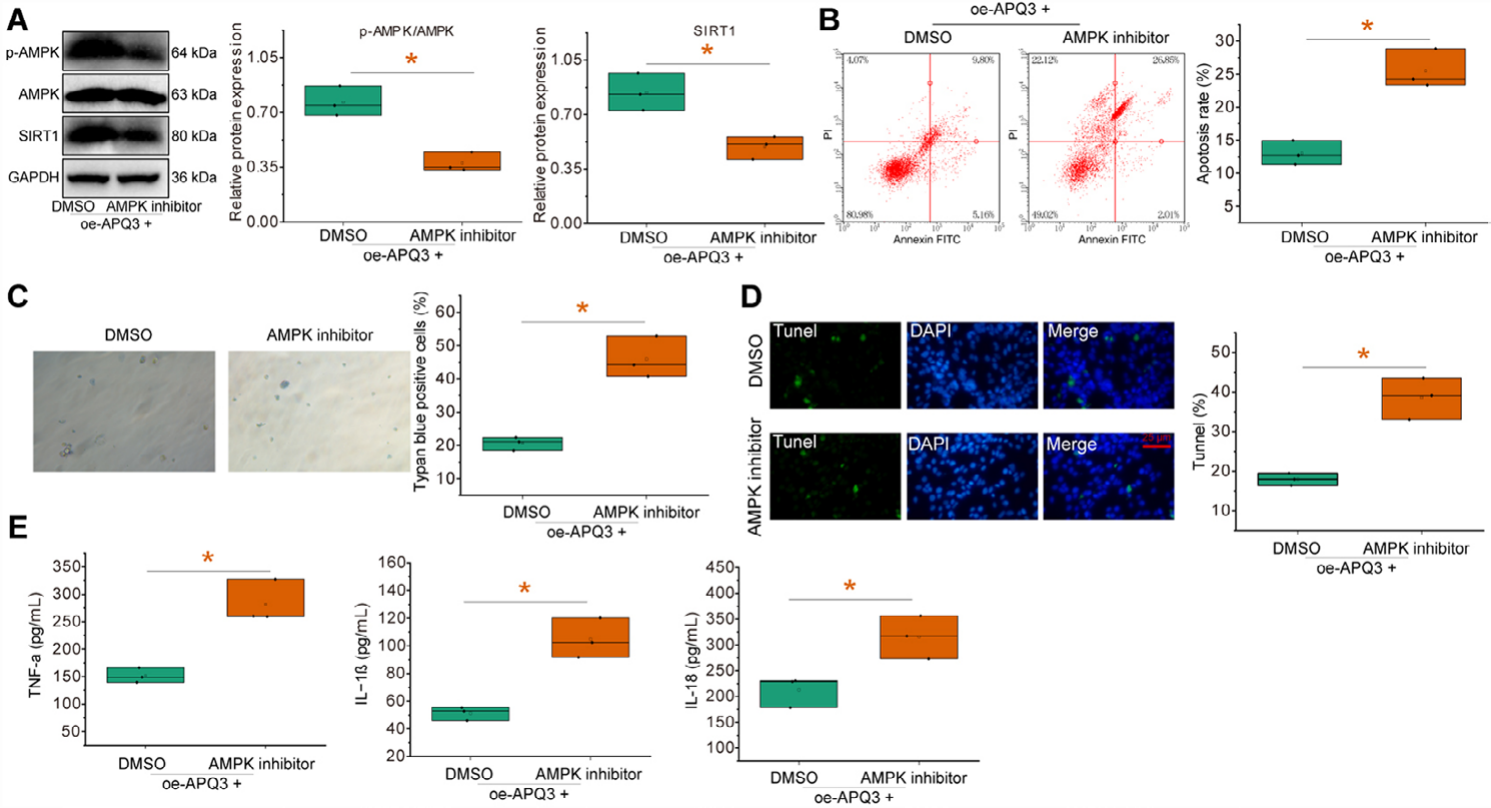
Effects of *AQP3* on LPS-induced inflammatory injury of gallbladder mucosal epithelial cells by regulating the *AMPK*/*SIRT1* signaling pathway. LPS-induced gallbladder mucosal epithelial cells were treated with or-*AQP3* alone or combined with an *AMPK* inhibitor. **A** Western blot to detect the protein expression and phosphorylation level of *AMPK* and protein expression of *SIRT1* in gallbladder mucosal epithelial cells. **B**, Flow cytometry to detect inflammatory injury of gallbladder mucosal epithelial cells. **C**, TUNEL staining to detect inflammatory injury of gallbladder mucosal epithelial cells. **D**, Trypan blue staining to observe the pyroptosis of gallbladder mucosal epithelial cells. **E**, ELISA to detect the expression of TNF-α, IL-1β, and IL-18 in gallbladder mucosal epithelial cells. All cell experiments were repeated three times. * *p* < 0.05

Compared with those upon oe-*AQP3* alone, there were no significant changes in the mRNA expression of *AMPK* or *SIRT1* in gallbladder mucosal epithelial cells in the presence of oe-*AQP3* + *AMPK* inhibitor. However, the protein expression and phosphorylation level of *AMPK* and protein expression of *SIRT1* were significantly reduced (Fig. 6A).

In addition, the number of inflammation-injured gallbladder mucosal epithelial cells with *AQP3* overexpression was significantly increased by additional inhibition of *AMPK*, accompanied by notable increases in the expression of TNF-α, IL-1β, and IL-18 (Fig. 6B-E).

The above results suggested that *AQP3* inhibited LPS-induced inflammatory injury of gallbladder mucosal epithelial cells through *AMPK*/*SIRT1* signaling pathway activation, while inactivation of the pathway reversed its effect.

### Overexpression of *AQP3* reduced gallbladder injury in mice with cholelithiasis through activation of the *AMPK*/*SIRT1* signaling pathway

**Fig. 7:**
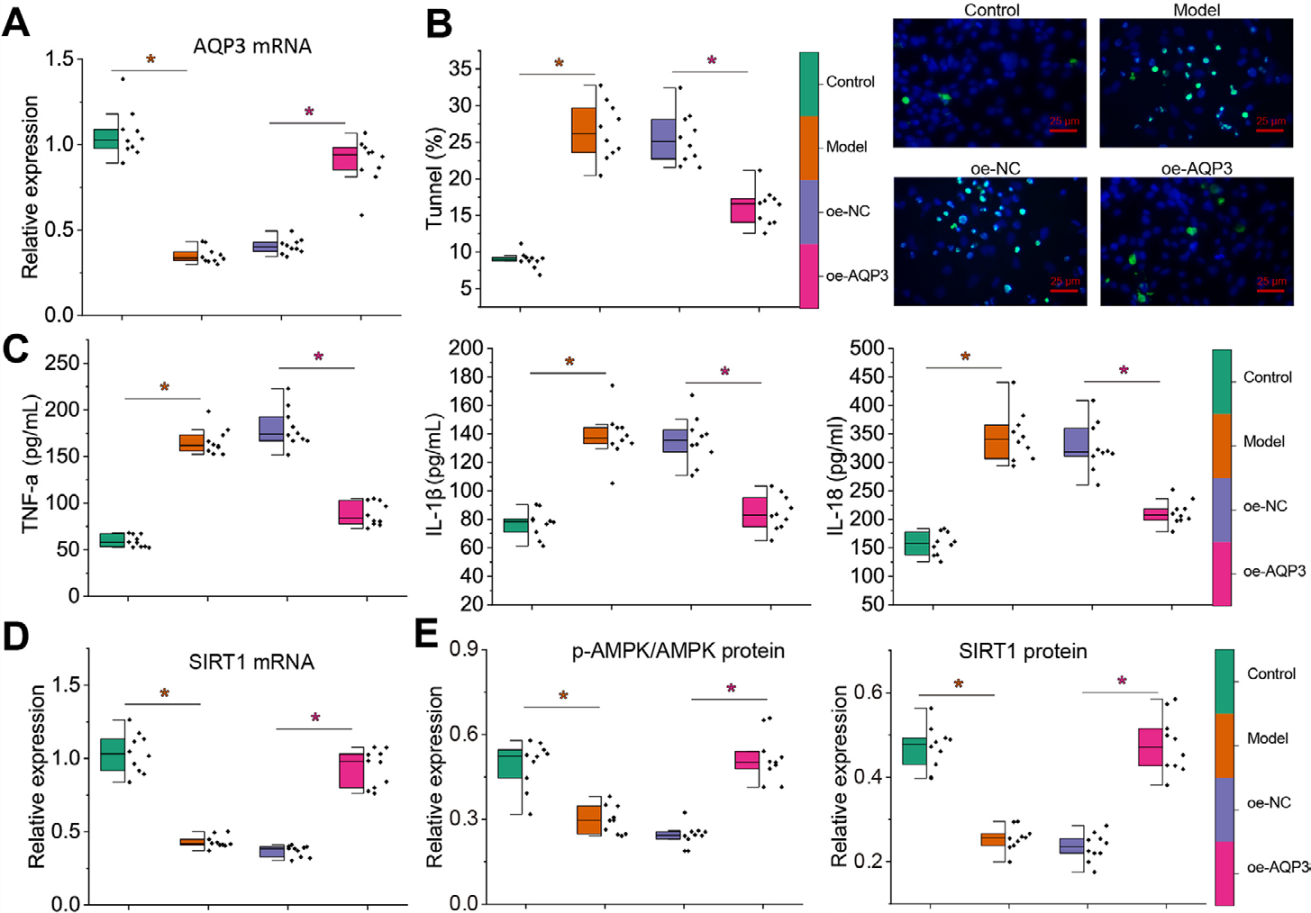
Effects of *AQP3* on mice with cholelithiasis by regulating the *AMPK*/*SIRT1* signaling pathway. **A**, RT-qPCR to detect the expression of *AQP3* in the gallbladder tissues of mice with cholelithiasis. **B**, TUNEL staining to detect the pyroptosis of gallbladder mucosal epithelial cells in mice with cholelithiasis after *AQP3* overexpression. Red fluorescence represents TUNEL, **C**, ELISA to detect the expression of TNF-α, IL-1β, and IL-18 in the gallbladder tissues of mice with cholelithiasis after *AQP3* overexpression. **D**, RT-qPCR to detect the mRNA expression of *SIRT1* in the gallbladder tissues of mice with cholelithiasis after *AQP3* overexpression. **E**, Western blot to detect the protein expression and phosphorylation level of *AMPK* and protein expression of *SIRT1* in the gallbladder tissues of mice with cholelithiasis after *AQP3* overexpression. n = 10. * *p* < 0.05

To further investigate the effect of *AQP3* on gallbladder injury in vivo, we constructed a mouse model of cholelithiasis overexpressing *AQP3*. The mRNA expression of *AQP3* was notably down-regulated in the gallbladder tissues of mice with cholelithiasis, which could be increased after transduction with a lentiviral vector harboring oe-*AQP3* (Fig. 7A). Furthermore, TUNEL staining revealed that compared with normal control mice, epithelial cell pyroptosis was increased in the gallbladder tissues of the mice with cholelithiasis. At the same time, overexpression of *AQP3* reduced the level of epithelial cell pyroptosis in gallbladder tissues (Fig. 7B).

Meanwhile, ELISA results showed that TNF-α, IL-1β and IL-18 expression was significantly up-regulated in gallbladder tissues of the mice with cholelithiasis relative to that in the normal control mice; overexpression of *AQP3* inhibited the expression of TNF-α, IL-1β, and IL-18 in gallbladder tissues of the mice with cholelithiasis (Fig. 7C). Moreover, the mRNA expression of *SIRT1* and phosphorylation levels of *SIRT1* and *AMPK* were significantly reduced in the gallbladder tissues of the mice with cholelithiasis relative to that in the normal control mice. Overexpression of *AQP3* activated the *AMPK*/*SIRT1* signaling pathway. It increased the mRNA expression of *AMPK*, protein expression and phosphorylation level of *AMPK*, and protein expression of *SIRT1* in the gallbladder tissues of the mice with cholelithiasis (Fig. 7D, E).

The above results indicated that overexpression of *AQP3* inhibited LPS-induced inflammatory injury of mouse gallbladder mucosal epithelial cells and alleviated gallbladder injury in mice via activation of the *AMPK*/*SIRT1* signaling pathway.

## Discussion

Gallstones can damage the gallbladder epithelium and induce chronic inflammation (Rosa et al. 2020). Based on high-throughput sequencing, this study explored the possible molecular mechanism by which *AQP3* affected gallstone formation in cholelithiasis. Our results revealed that *AQP3* could inhibit the inflammatory injury of gallbladder mucosal epithelial cells by activating the *AMPK*/*SIRT1* signaling pathway.

Our study found that *AQP3* was significantly downregulated in mice with cholelithiasis and that *AQP3* might have an important role in LPS-induced gallbladder mucosal injury. AQP family proteins may be protective proteins that could alleviate inflammatory injury of the gallbladder mucosa epithelium of mice with cholelithiasis, but the mechanism needs further investigation (Kube et al. 2022). Our in vivo animal studies revealed that *AQP3* is a negative regulator of inflammatory injury of the mucosal epithelium of the gallbladder in mice with cholelithiasis and is a potential target for treating cholelithiasis. Notably, there is a scarcity of reports regarding the role of *AQP3* in cholelithiasis. The relationship between *AQP3* and inflammatory response has also been reported. For instance, rescued *AQP3* by pterostilbene might aid in producing anti-inflammation in keratinocytes (Teng et al. 2021). Reduced expression of *AQP3* was observed after LPS administration in the mouse kidney (Yu et al. 2018). In addition, previous studies have also reported a close relationship between *AQP3* and mucosal injury. In the occurrence of mucosal injury, upregulation of *AQP3* expression could make compensation for the mucosal injury and delay the disease progression of oral lichen planus (Agha-Hosseini et al. 2020). Our findings are consistent with the studies mentioned above and suggest that *AQP3* may be involved in LPS-induced gallbladder mucosal injury in mice with cholelithiasis.

Mechanistically, we found that overexpression of *AQP3* attenuated the LPS-induced inflammatory injury of gallbladder mucosal epithelial cells by activating the *AMPK*/*SIRT1* signaling pathway. The *AMPK* signaling pathway may be associated with water metabolism (Zhao et al. 2017), and ketoglutarate could increase water homeostasis by up-regulating *AMPK* pathway-related genes, including *AMPK*α1, *AMPK*α2, and *SIRT1* and elevate the expression of AQPs such as *AQP3* in LPS-challenged piglets (He et al. 2017), indicating the possible interaction between *AQP3* as a water channel and the *AMPK* signaling pathway. Intriguingly, prior research has suggested the role of the *AMPK*/*SIRT1* signaling pathway in gallstone formation. For instance, activated *AMPK* signaling by caveolin-1 aided in diminishing expression of gallbladder mucin-1 and MUC5ac and accumulation of gallbladder cholesterol, thereby conferring prevention against cholesterol gallstone disease (Li et al. 2022). Moreover, the reduced phosphorylation level of *AMPK*α2 was observed in gallbladder stones and primary intrahepatic bile duct stones, and leptin-mediated *AMPK*α2 could affect cholelithiasis by regulating bile acid metabolism (Wen et al. 2021). In addition, hepatic deprivation of *SIRT1* could contribute to cholesterol gallstone formation in mice by decreasing hepatocyte nuclear factor 1α/farnesoid X receptor signaling (Purushotham et al. 2012). Accumulating evidence has highlighted the alleviatory function of the *AMPK*/*SIRT1* signaling pathway on inflammatory injury of epithelial cells, such as intestinal epithelial cells (Zhang & Wu, 2021), pulmonary epithelial cells (Li et al. 2019) and gastric epithelial cells (Peng & Liu, 2022). These studies supported our findings that overexpression of *AQP3* ameliorated gallbladder injury by activating the *AMPK*/*SIRT1* signaling pathway.

## Conclusion

**Fig. 8:**
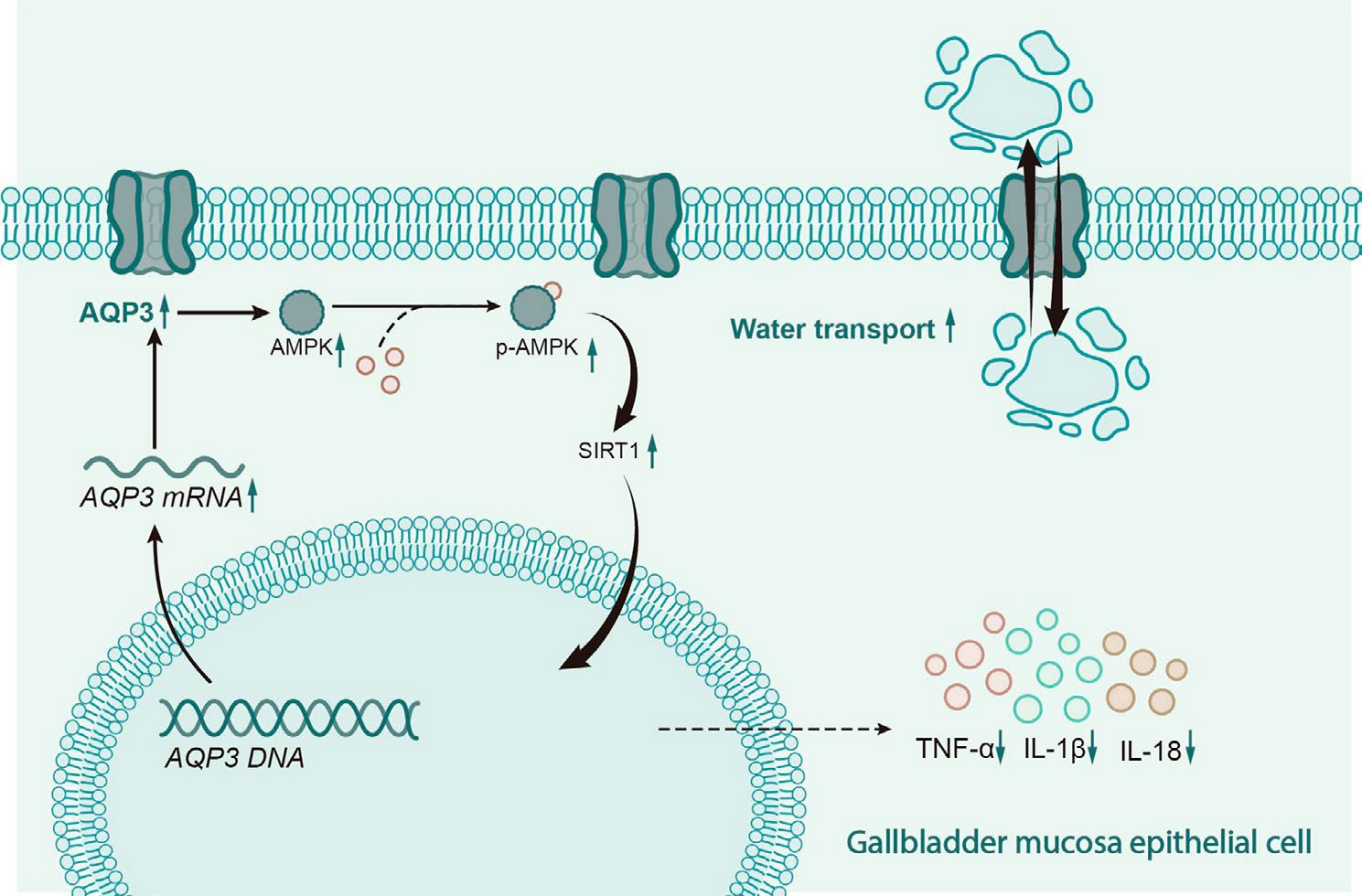
Molecular mechanism of *AQP3* in cholelithiasis *via* activation of *AMPK*/*SIRT1* signaling pathway. *AQP3* induces the activation of the *AMPK*/*SIRT1* signaling pathway, which can reduce the inflammatory injury of gallbladder mucosal epithelial cells and improve the water transport inside and outside the mucosa. By this mechanism, the gallbladder damage of cholelithiasis can be reduced

Based on the above results, we could conclude that *AQP3* could activate the *AMPK*/*SIRT1* signaling pathway, which reduced inflammatory injury of the gallbladder mucosal epithelial cells, thereby alleviating gallbladder damage and preventing gallstone formation in mice (Fig. 8). Our study provides new insight and a theoretical basis for diagnosing and treating cholelithiasis. However, it also has some limitations. Firstly, although we observed the corresponding molecular changes and inflammatory damage in cholelithiasis model mice that overexpressed *AQP3*, further exploration using knockout gene mice is necessary. Secondly, there are three methods to induce cholelithiasis in mice: the high cholesterol plus bile acid method, the high bile acid method, and the deoxycholic acid method. Our study only used the high cholesterol method, and further research using various modeling methods and clinical studies is needed to understand the crucial role of *AQP3* in cholelithiasis. A previous study has reported that *AQP3* may mediate triglyceride synthesis by transporting glycerol or inhibit inflammation by transporting peroxides, which may induce activation of the *AMPK*/*SIRT1* signaling pathway (E et al. 2021). We did not further explore this in our current study, which is another limitation. In addition, due to budget constraints, this study could not confirm the relationship of *AQP3* with gallbladder epithelial cell necrosis in gene-knockout mouse models. Moreover, AKG plays a crucial role in regulating water and ion homeostasis by regulating the *AMPK* pathway; this study did not investigate the relationship between the transport function of water, glycerol, and peroxide mediated by *AQP3* and the *AMPK*/*SIRT1* signaling pathway. Therefore, further research is needed to explore and verify the mechanism of action of *AQP3* in gallstone formation.

### Electronic supplementary material

Below is the link to the electronic supplementary material.

Supplementary Material 1

Supplementary Material 2

## Data Availability

The data supporting this study’s findings are available on request from the corresponding author.

## Abbreviations

*AQP3*: Aquaporin 3
DEG: Differentially expressed gene
LPS: Lipopolysaccharide
PPI: Protein-protein interaction
NC: Negative control

## References

[CR1] Agha-Hosseini F, Barati H, Moosavi MS (2020). Aquaporin3 (*AQP3*) expression in oral epithelium in oral lichen planus. Exp Mol Pathol.

[CR2] Alves-Fernandes DK, Jasiulionis MG (2019). The role of *SIRT1* on DNA damage response and epigenetic alterations in Cancer. Int J Mol Sci.

[CR3] Chen L, Yang H, Li H, He C, Yang L, Lv G (2022). Insights into modifiable risk factors of cholelithiasis: a mendelian randomization study. Hepatology.

[CR4] Dalsgaard T, Cecchi CR, Askou AL, Bak RO, Andersen PO, Hougaard D (2018). Improved Lentiviral Gene Delivery to Mouse Liver by Hydrodynamic Vector Injection through tail vein. Mol Ther Nucleic Acids.

[CR6] Franke M, Bieber M, Kraft P, Weber ANR, Stoll G, Schuhmann MK (2021). The NLRP3 inflammasome drives inflammation in ischemia/reperfusion injury after transient middle cerebral artery occlusion in mice. Brain Behav Immun.

[CR7] Gong L, Tang Y, An R, Lin M, Chen L, Du J (2017). RTN1-C mediates cerebral ischemia/reperfusion injury via ER stress and mitochondria-associated apoptosis pathways. Cell Death Dis.

[CR8] Guo L, Wang D, Alexander HY, Ren X, Ma H (2022). Long non-coding RNA H19 contributes to spinal cord ischemia/reperfusion injury through increasing neuronal pyroptosis by miR-181a-5p/HMGB1 axis. Aging (Albany NY).

[CR10] He L, Huang N, Li H, Tian J, Zhou X, Li T (2017). *AMPK*/alpha-Ketoglutarate Axis regulates Intestinal Water and Ion Homeostasis in Young Pigs. J Agric Food Chem.

[CR9] He C, Shen W, Chen C, Wang Q, Lu Q, Shao W (2021). Circadian rhythm disruption influenced hepatic lipid metabolism, gut microbiota and promoted cholesterol gallstone formation in mice. Front Endocrinol (Lausanne).

[CR11] Hu H, Shao W, Liu Q, Liu N, Wang Q, Xu J (2022). Gut microbiota promotes cholesterol gallstone formation by modulating bile acid composition and biliary cholesterol secretion. Nat Commun.

[CR12] Huang Y, Yang M, Yang H, Zeng Z (2010). Upregulation of the GRIM-19 gene suppresses invasion and metastasis of human gastric cancer SGC-7901 cell line. Exp Cell Res.

[CR13] Huang YF, Gu CJ, Wang Q, Xu L, Chen J, Zhou W (2020). The protective effort of GPCR kinase 2-interacting protein-1 in neurons via promoting Beclin1-Parkin induced mitophagy at the early stage of spinal cord ischemia-reperfusion injury. FASEB J.

[CR14] Imamura H, Adachi T, Kitasato A, Sakai Y, Ono S, Hara T (2017). A modified method for purifying gallbladder epithelial cells using fluorescence-activated cell sorting. In Vivo.

[CR15] Jeyaseelan K, Sepramaniam S, Armugam A, Wintour EM (2006). Aquaporins: a promising target for drug development. Expert Opin Ther Targets.

[CR16] Kube I, Kowalczyk M, Hofmann U, Ghallab A, Hengstler JG, Fuhrer D (2022). Hepatobiliary thyroid hormone Deficiency Impacts bile Acid Hydrophilicity and Aquaporins in Cholestatic C57BL/6J mice. Int J Mol Sci.

[CR17] Lali FV, Hunt AE, Turner SJ, Foxwell BM (2000). The pyridinyl imidazole inhibitor SB203580 blocks phosphoinositide-dependent protein kinase activity, protein kinase B phosphorylation, and retinoblastoma hyperphosphorylation in interleukin-2-stimulated T cells independently of p38 mitogen-activated protein kinase. J Biol Chem.

[CR18] Lammert F, Gurusamy K, Ko CW, Miquel JF, Mendez-Sanchez N, Portincasa P (2016). Gallstones Nat Rev Dis Primers.

[CR19] Li L, Zhang H, Ma T, Verkman AS (2009). Very high aquaporin-1 facilitated water permeability in mouse gallbladder. Am J Physiol Gastrointest Liver Physiol.

[CR21] Li X, Jamal M, Guo P, Jin Z, Zheng F, Song X (2019). Irisin alleviates pulmonary epithelial barrier dysfunction in sepsis-induced acute lung injury via activation of *AMPK*/*SIRT1* pathways. Biomed Pharmacother.

[CR20] Li S, Chen H, Jiang X, Hu F, Li Y, Xu G (2022). Adeno-associated virus-based caveolin-1 delivery via different routes for the prevention of cholesterol gallstone formation. Lipids Health Dis.

[CR22] Lim JH, Kim DH, Han DW, Kwak JY, Bae HR (2016). The effect of *AQP3* deficiency on fuel selection during a single bout of exhausting exercise. Pflugers Arch.

[CR23] Liu X, Lv Y, Xie Y, Hong Q, Cai G, Zhang S (2011). Change of MAX interactor 1 expression in an anti-Thy1 nephritis model and its effect on mesangial cell proliferation. Cell Physiol Biochem.

[CR24] Liu Z, Yu Y, Huang Z, Kong Y, Hu X, Xiao W (2019). CircRNA-5692 inhibits the progression of hepatocellular carcinoma by sponging mir-328-5p to enhance DAB2IP expression. Cell Death Dis.

[CR25] Lu X, Lv C, Zhao Y, Wang Y, Li Y, Ji C (2022). TSG-6 released from adipose stem cells-derived small extracellular vesicles protects against spinal cord ischemia-reperfusion injury by inhibiting endoplasmic reticulum stress. Stem Cell Res Ther.

[CR26] Mattos DR, Wan X, Serrill JD, Nguyen MH, Humphreys IR, Viollet B (2022). The Marine-Derived Macrolactone Mandelalide A is an indirect activator of *AMPK*. Mar Drugs.

[CR27] Maurer KJ, Rao VP, Ge Z, Rogers AB, Oura TJ, Carey MC (2007). T-cell function is critical for murine cholesterol gallstone formation. Gastroenterology.

[CR28] Maurya SK, Herrera JL, Sahoo SK, Reis FCG, Vega RB, Kelly DP (2018). Sarcolipin Signaling promotes mitochondrial Biogenesis and oxidative metabolism in skeletal muscle. Cell Rep.

[CR29] Mobasheri A, Marples D (2004). Expression of the AQP-1 water channel in normal human tissues: a semiquantitative study using tissue microarray technology. Am J Physiol Cell Physiol.

[CR30] Moschetta A, Bookout AL, Mangelsdorf DJ (2004). Prevention of cholesterol gallstone disease by FXR agonists in a mouse model. Nat Med.

[CR31] Movahed M, Brockie S, Hong J, Fehlings MG (2021). Transcriptomic Hallmarks of Ischemia-Reperfusion Injury. Cells.

[CR32] O’Leary CE, Sbierski-Kind J, Kotas ME, Wagner JC, Liang HE, Schroeder AW (2022). Bile acid-sensitive tuft cells regulate biliary neutrophil influx. Sci Immunol.

[CR34] Peng ZT, Liu H (2022). Puerarin attenuates LPS-induced inflammatory injury in gastric epithelial cells by repressing NLRP3 inflammasome-mediated apoptosis. Toxicol In Vitro.

[CR33] Peng Z, Li M, Tan X, Xiang P, Wang H, Luo Y (2020). Mir-211-5p alleviates focal cerebral ischemia-reperfusion injury in rats by down-regulating the expression of COX2. Biochem Pharmacol.

[CR35] Purushotham A, Xu Q, Lu J, Foley JF, Yan X, Kim DH (2012). Hepatic deletion of *SIRT1* decreases hepatocyte nuclear factor 1alpha/farnesoid X receptor signaling and induces formation of cholesterol gallstones in mice. Mol Cell Biol.

[CR36] Rosa L, Lobos-Gonzalez L, Munoz-Durango N, Garcia P, Bizama C, Gomez N (2020). Evaluation of the chemopreventive potentials of ezetimibe and aspirin in a novel mouse model of gallbladder preneoplasia. Mol Oncol.

[CR37] Teng WL, Huang PH, Wang HC, Tseng CH, Yen FL (2021). Pterostilbene attenuates Particulate Matter-Induced oxidative stress, inflammation and aging in keratinocytes. Antioxid (Basel).

[CR38] Wei L, Li J, Han Z, Chen Z, Zhang Q (2019). Silencing of lncRNA MALAT1 prevents inflammatory Injury after Lung Transplant ischemia-reperfusion by downregulation of IL-8 via p300. Mol Ther Nucleic Acids.

[CR39] Wen J, Jiang Y, Lei Z, He J, Ye M, Fu W (2021). Leptin influence cholelithiasis formation by regulating bile acid metabolism. Turk J Gastroenterol.

[CR40] Wu S, Zou MH (2020). AMPK, mitochondrial function, and Cardiovascular Disease. Int J Mol Sci.

[CR41] Yu G, Liu Q, Dong X, Tang K, Li B, Liu C (2018). Inhibition of inflammation using diacerein markedly improved renal function in endotoxemic acute kidney-injured mice. Cell Mol Biol Lett.

[CR42] Zhang YJ, Wu Q (2021). Sulforaphane protects intestinal epithelial cells against lipopolysaccharide-induced injury by activating the *AMPK*/*SIRT1*/PGC-1a pathway. Bioengineered.

[CR43] Zhao HW, Li YW, Feng R, Yu JB, Li J, Zhang Y (2015). TGF-beta/Smad2/3 signal pathway involves in U251 cell proliferation and apoptosis. Gene.

[CR44] Zhao WX, Cui N, Jiang HQ, Ji XM, Han XC, Han BB (2017). Effects of Radix Astragali and its Split Components on Gene expression profiles related to Water Metabolism in rats with the dampness stagnancy due to spleen Deficiency Syndrome. Evid Based Complement Alternat Med.

