# Supplementary figures and images for: AQP3-mediated activation of the AMPK/SIRT1 signaling pathway curtails gallstone formation in mice by inhibiting inflammatory injury of gallbladder mucosal epithelial cells
Source: Mol Med. 2023 Aug 28;29:116. doi: 10.1186/s10020-023-00712-8 (PMC10463418; doi:10.1186/s10020-023-00712-8)

Figure 1:


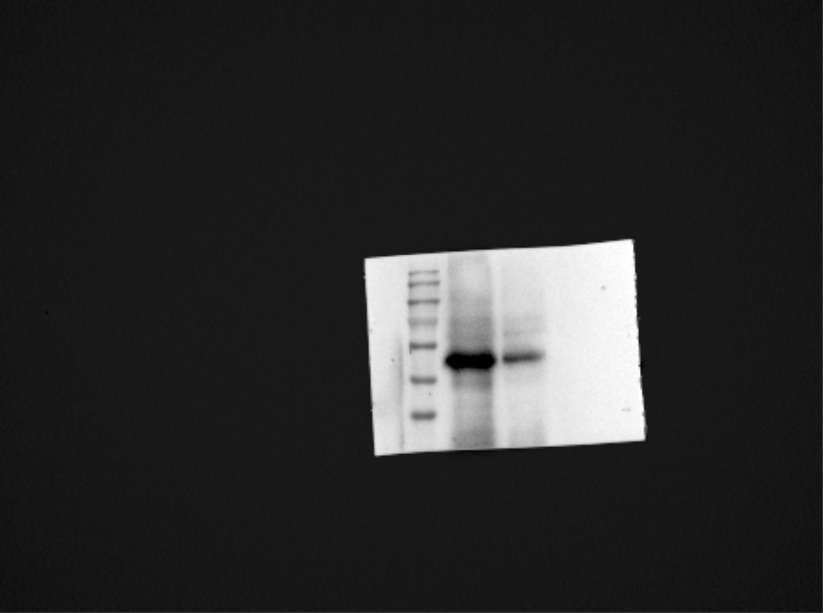
 1G-1


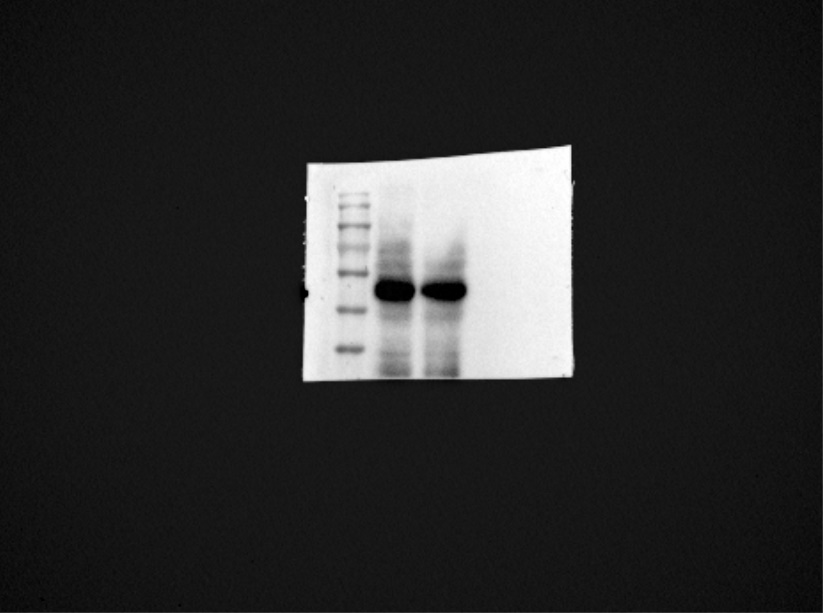
 1G-2

Figure 5:


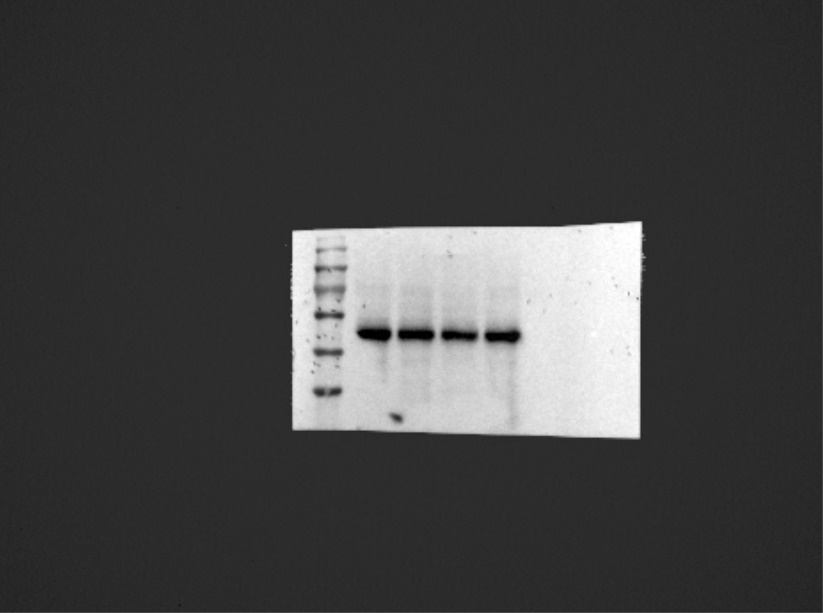
 5B-1


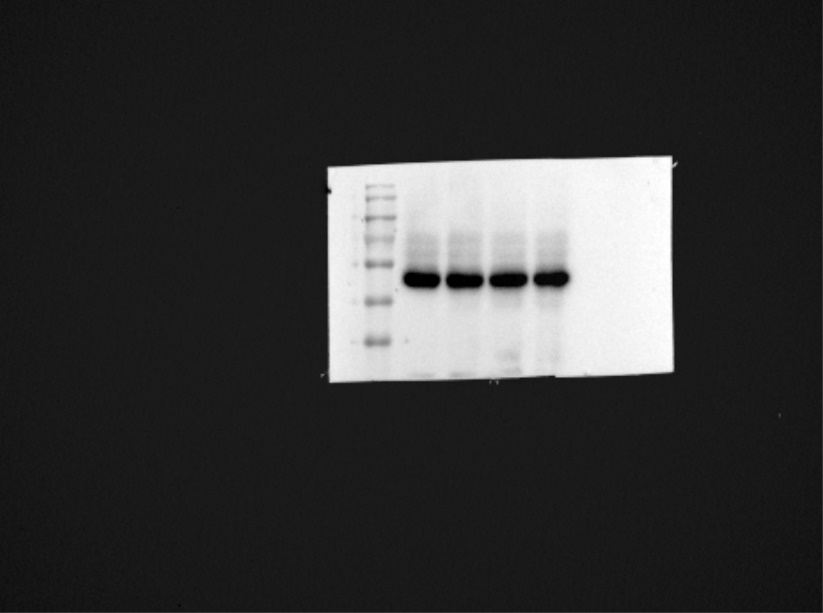
 5B-2


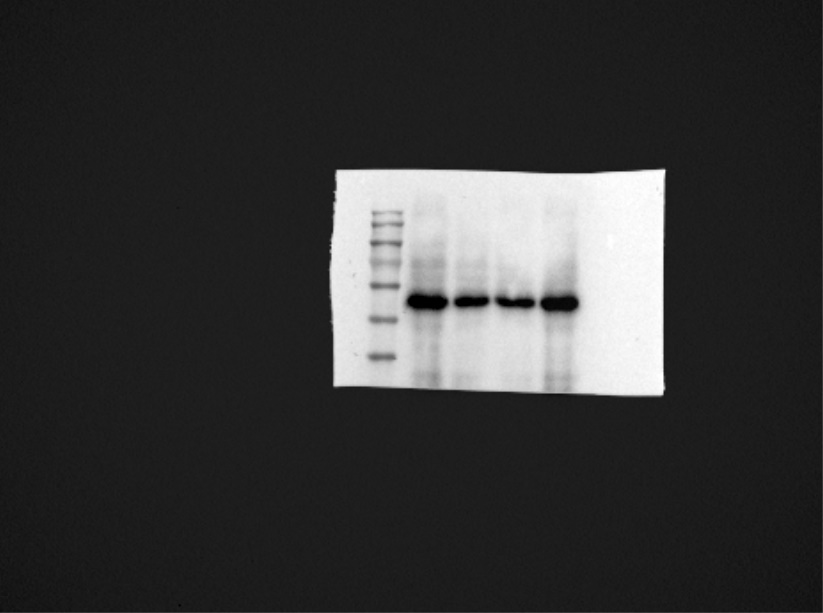
 5B-3


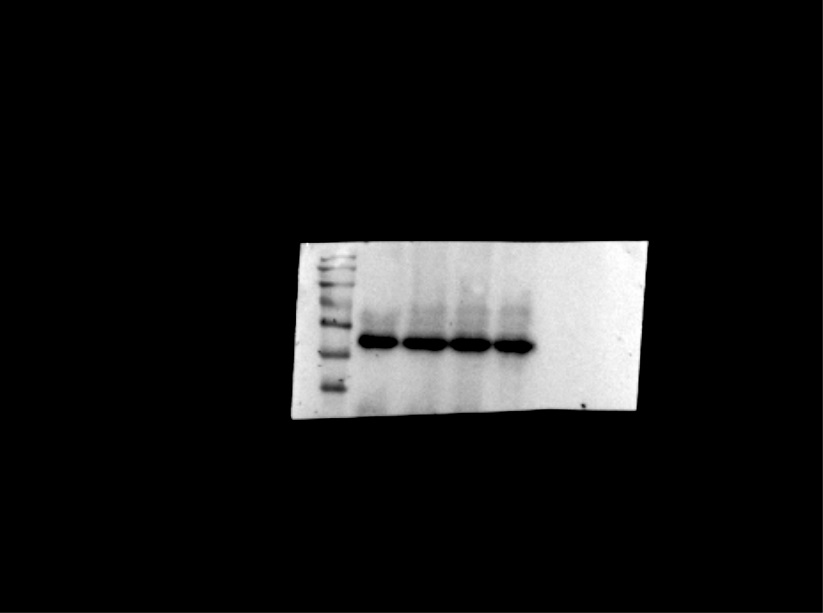
 5B-4

Figure 6:


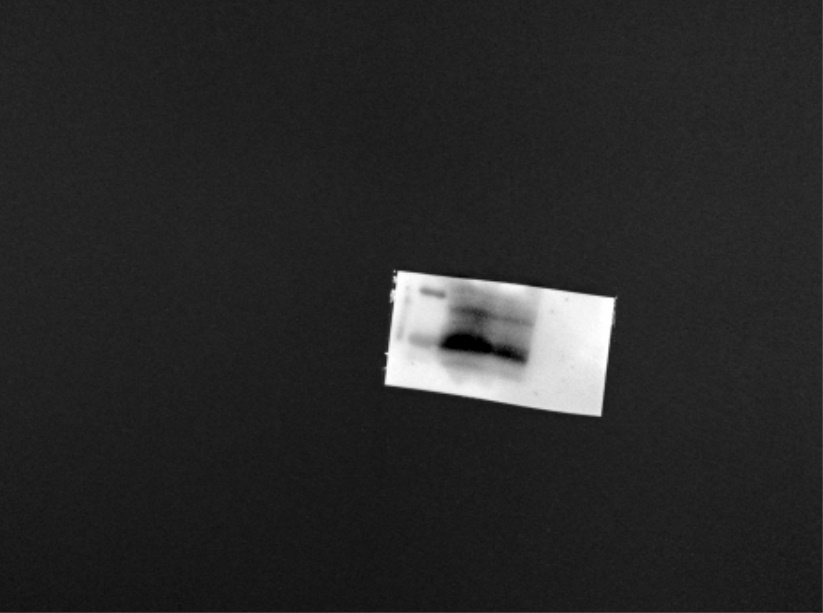
 6A-1


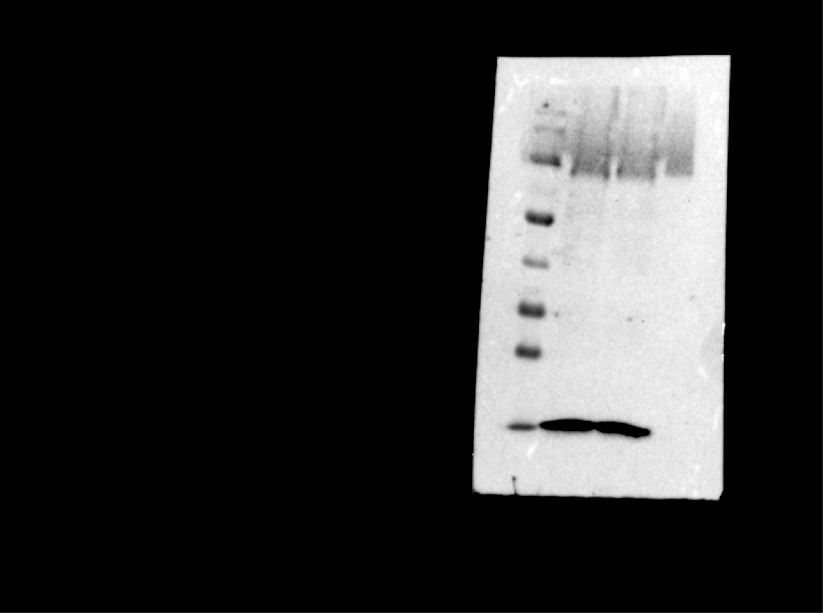
 6A-2


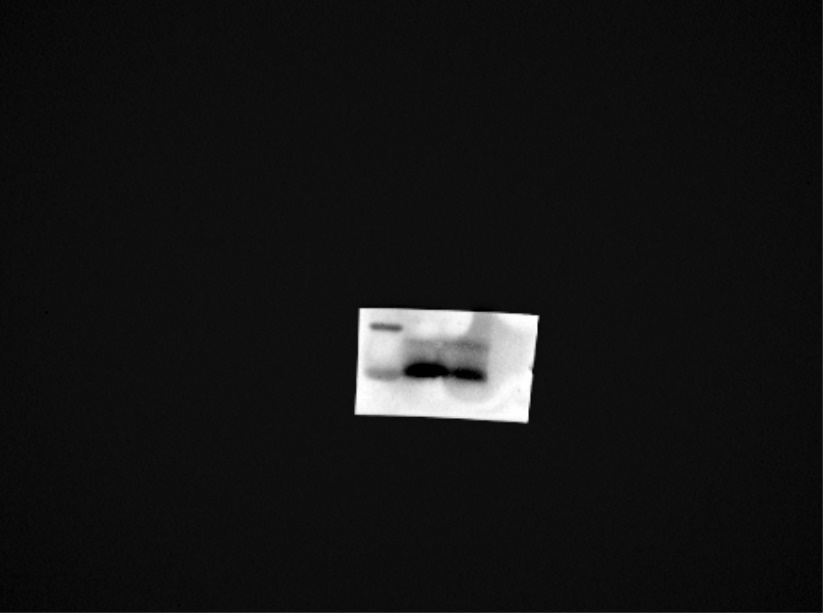
 6A-3


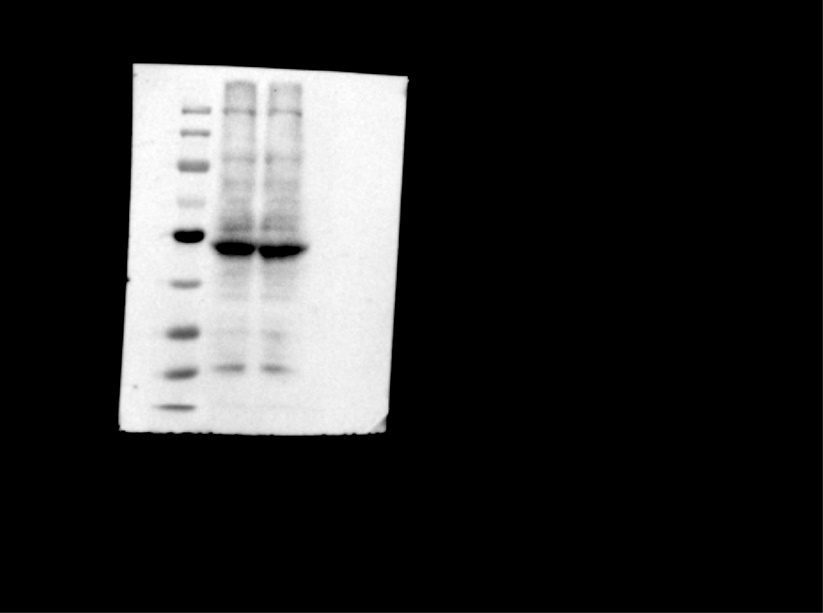
 6A-4

Supplement: Supplementary file 1 — Supplementary Material 1 [file 10020_2023_712_MOESM1_ESM.docx]
