# Supplementary material for: AQP3-mediated activation of the AMPK/SIRT1 signaling pathway curtails gallstone formation in mice by inhibiting inflammatory injury of gallbladder mucosal epithelial cells
Source: Mol Med. 2023 Aug 28;29:116. doi: 10.1186/s10020-023-00712-8 (PMC10463418; doi:10.1186/s10020-023-00712-8)
Supplement: Supplementary file 2 — Supplementary Material 2 [file 10020_2023_712_MOESM2_ESM.docx]

**Table S1** Primer sequences for RT-qPCR

| Gene | Sequence (5’-3’) |
| --- | --- |
| AQP3 (mouse) | Forward: 5'-TTTGGCTTCGCTGTCACCCTTG-3' |
|  | Reverse: 5'-CCAGTGCATAGATGGGCAGCTT-3' |
| SIRT1 (mouse) | Forward: 5'-CGGCTACCGAGGTCCATATAC-3' |
|  | Reverse: 5'-CTGCAACCTGCTCCAAGGTA-3' |
| GAPDH (mouse) | Forward: 5'-CATCACTGCCACCCAGAAGACTG-3' |
|  | Reverse: 5'-ATGCCAGTGAGCTTCCCGTTCAG-3' |

Note: RT-qPCR, reverse transcription polymerase chain reaction; AQP3, aquaporin 3; SIRT1, sirtuin 1; GAPDH, glyceraldehyde-3-phosphate dehydrogenase.**Table S2** Manufacturer information of primary antibodies

| Antibody | Cat. | Manufacturer | Country |
| --- | --- | --- | --- |
| Anti-AQP3 | ab125219 | Abcam | UK |
| Anti-AMPK | ab32047 | Abcam | UK |
| Anti-p-AMPK | ab92701 | Abcam | UK |
| Anti-SIRT1 | ab189494 | Abcam | UK |
| Anti-GAPDH | ab8245 | Abcam | UK |

Note: AQP3, aquaporin 3; p-, phosphorylated-; AMPK, adenosine monophosphate-activated protein kinase; SIRT1, sirtuin 1; GAPDH, glyceraldehyde-3-phosphate dehydrogenase.
